# Saikosaponin D Inducing Apoptosis and Autophagy through the Activation of Endoplasmic Reticulum Stress in Glioblastoma

**DOI:** 10.1155/2022/5489553

**Published:** 2022-11-24

**Authors:** Guimei Liu, Yuehong Guan, Yongxian Liu, Yaping Wang, Jing Zhang, Yusi Liu, Xiaobin Liu

**Affiliations:** Department of Medical Immunology, College of Basic Medical Sciences, Yan'an University, Yan'an 716000, China

## Abstract

Saikosaponin D (SSD), a saponin derivative, is extracted from *Bupleurum falcatum*. It exhibits an inhibitory effect on a number of tumor cells and is relatively safe when used at therapeutic doses. However, its effects on glioblastoma multiforme (GBM) have not been fully explored. This study is aimed at investigating the cytotoxic effects of SSD in GBM cell lines. SSD induces apoptosis and autophagy by activating endoplasmic reticulum (ER) stress in GBM cells. GBM cell proliferation activity and morphology were observed using the Cell Counting Kit-8 assay and hematoxylin and eosin staining. Hoechst 33258 fluorescence staining and flow cytometry were performed to assess apoptosis. Western blotting and immunocytochemical staining were used to detect protein expression and distribution. SSD significantly inhibited the proliferation of RG-2, U87-MG, and U251 cells in a dose-dependent manner, and the proportion of apoptotic cells increased significantly. Additionally, the expressions of ER-, apoptosis-, and autophagy-related proteins were significantly upregulated and distributed in the cytoplasm and nucleus. Therefore, SSD may be considered a novel treatment option for GBM. This study demonstrated the anti-GBM effect of SSD from the perspectives of cell apoptosis and autophagy.

## 1. Introduction

Gliomas are primary brain tumors. The most common types of gliomas are astrocytomas, oligodendrogliomas, and ependymomas. The World Health Organization classifies malignant gliomas into grades I, II, III, and IV based on their pathological characteristics, nuclear atypia, mitotic activity, vascular proliferation, necrosis, proliferative potential, clinical course, and treatment outcomes [1, 2]. Grades I and II are low-grade diffuse gliomas, most of which eventually develop into high-grade gliomas; and grades III and IV are high-grade gliomas, such as glioblastoma multiforme (GBM), which are associated with a low differentiation ability, the highest degree of malignancy, aggressive growth, and a poor prognosis. The incidence rate of GBM is 46.1%. The average survival time of patients is approximately 14.6 months, and the 5-year survival rate is <5%. Routine surgical treatment cannot completely remove GBM tissue, and residual tumors are less sensitive to radiotherapy and chemotherapy. Therefore, the recurrence rate of GBM is high, which significantly affects the prognosis [3]. Surgery is currently the mainstay of treatment for GBM, followed by radiotherapy and chemotherapy. However, owing to the inability to cross the blood-brain barrier and drug resistance, several anticancer drugs are less effective in patients with GBM. Therefore, new anti-GBM drugs with low toxicity and high efficiency are urgently required. Traditional Chinese medicine is effective in improving the symptoms of cancer and prolonging survival time [4].

**Figure 1:**
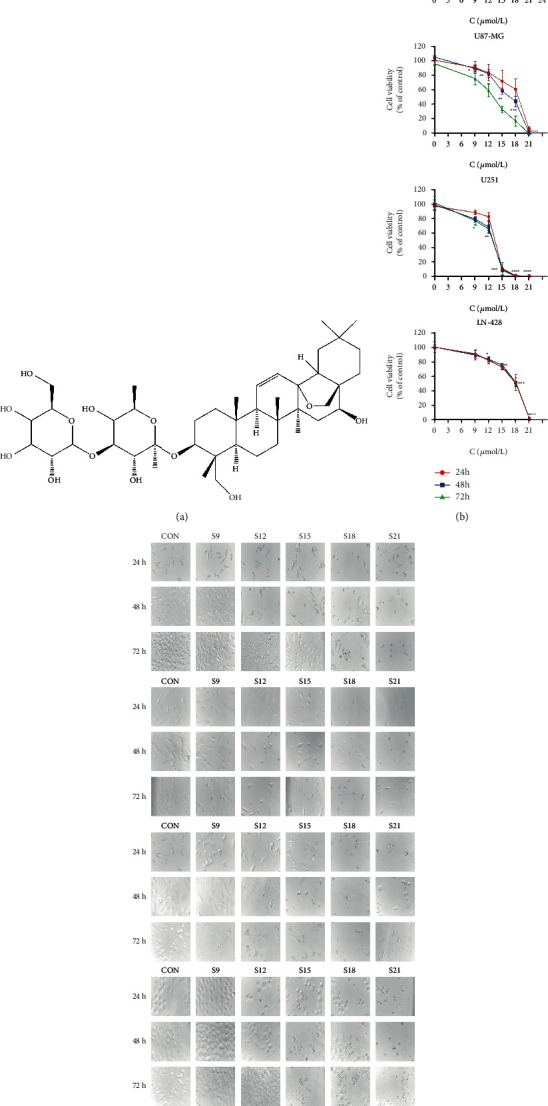
SSD inhibited the proliferation of GBM cells and GBM cell morphology. Distinct cellular response of rat RG-2 GBM cell line, human GBM cell lines U87-MG, U251, and LN-428 were to 9 *μ*M (S9), 12 *μ*M (S12), 15 *μ*M (S15), 18 *μ*M (S18), and 21 *μ*M (S21) SSD treatment for 24, 48, and 72 h. (a) Structure of Saikosaponin D. (b) CCK-8 cell proliferation assay. (c) The changes in cell morphology (40× magnification) observed by the inverted microscope. ^∗^*P* < 0.05, ^∗∗^*P* < 0.01, ^∗∗∗^*P* < 0.001, and ^∗∗∗∗^*P* < 0.0001 vs. CON group. The error bars, the mean ± standard deviation.

Saikosaponin D (SSD) (Figure 1(a)) is a saponin derivative extracted from the rhizome of the natural plant Radix Bupleuri (RB). It is used to treat influenza, hyperlipidemia, menstrual problems, stress, hepatitis, and depression. It inhibits certain tumors, such as liver cancer [5, 6], lung cancer [7, 8], and melanoma [9], and it is relatively safe when used at therapeutic doses. Furthermore, Du et al. found that Saikosaponin A, which is also isolated from RB, could be an efficient anticancer drug owing to its ability to inhibit the growth of cervical tumors through the activation of endoplasmic reticulum (ER) stress [10].

As any perturbation in ER function leads to the accumulation of unfolded or misfolded proteins, an unfolded protein response (UPR) is generally used to indicate the occurrence of ER stress [11, 12]. PKR-like ER kinase (PERK) and transcription factor 6 (ATF6) are major transducers of the ER stress response [13, 14]. When UPR is induced, the expression of glucose-regulated protein 78-kDa (GRP78) is promoted to alleviate or abort the ER stress response [15], thereby restoring homeostasis in the cellular environment. GRP78 is a key regulator of ER stress, and its upregulation is often used as a marker of ER stress. Cypermethrin can induce the expression of ER stress-related proapoptotic molecules, such as C/EBP homologous protein (CHOP) and Caspase-12, ultimately leading to apoptosis [16].

Although numerous studies have demonstrated the ability of SSD to suppress cell proliferation and promote apoptosis in many tumor cells, such as breast, lung, and liver cancer cells, the mechanism underlying the anti-GBM effect of SSD remains unknown. Therefore, the current study is aimed at investigating the anti-GBM effect of SSD and exploring its mechanism from the perspectives of cell apoptosis and autophagy, which can lay a foundation for further development of SSD and provide novel insights into the treatment of GBM.

## 2. Materials and Methods

### 2.1. Agents

SSD (purity ≥98%, high-performance liquid chromatography) was purchased from Shanghai Yuanye Bio-Technology (Shanghai, China). SSD was dissolved in dimethyl sulfoxide (Solarbio, Beijing, China) to prepare a stock solution at a concentration of 1 mM, wrapped in aluminum foil to protect against light, and stored at −20°C. The Cell Counting Kit-8 (CCK-8), Hoechst 33258 staining kit, Annexin V-FITC Apoptosis Kit, and Immunocytochemical staining (ICC) kit were purchased from BOSTER Biotechnology (Wuhan, China) and stored at 4°C. GRP78, CHOP, PERK, ATF6, Caspase-12, Caspase-9, Caspase-3, Beclin 1, *β*-actin, and peroxidase-conjugated anti-rabbit antibodies were purchased from Proteintech Group, Inc. (Wuhan, China). LC3 and poly (ADP-ribose) polymerase (PARP) antibodies were obtained from Abrasives Mart (Shanghai, China).

### 2.2. Cell Line and Culture

The rat RG-2 GBM cell line and human U87-MG, U251, and LN-428 GBM cell lines used in this study were provided by Professor Yusi Liu, Department of Immunology, Central Hospital of Yan'an University, Shanxi, China. GBM cells were cultured in Dulbecco's modified Eagle medium (Gibco, Grand Island, NY, USA) containing 10% fetal bovine serum (Gibco, Grand Island, NY, USA) and 1% penicillin-streptomycin (Gibco, Grand Island, NY, USA) and maintained at 37°C in a 5% CO_2_-humidified atmosphere.

### 2.3. Cell Viability Assay

RG-2, U87-MG, U251, and LN-428 cells (100 *μ*L, 3 × 10^3^ cells/well) were seeded into 96-well tissue culture plates. The four GBM cell lines were collected after treatment with different concentrations (0, 9, 12, 15, 18, and 21 *μ*M) of SSD for 24, 48, and 72 h. Subsequently, 10 *μ*L of the CCK-8 reagent was added to each well and incubated for an additional 1 h at 37°C. Absorbance was measured using a microplate reader at 452 nm. Subsequently, the cell viability and 50% inhibitory concentration (IC50) of SSD were calculated.

### 2.4. Cell Morphology

Cell morphology was assessed using hematoxylin and eosin (H&E) staining. The four GBM cell lines were treated with various concentrations (0, 9, 12, 15, 18, and 21 *μ*M) of SSD. After 24, 48, and 72 h, cells were observed under an inverted microscope (Nikon, China). H&E staining was then performed after selecting high and low concentrations of GBM cells according to the aforementioned results. Briefly, cells were plated in 6-well plates and treated with various concentrations (0, 9, and 15 *μ*M) of SSD for 48 h, fixed with 4% paraformaldehyde for 15 min, and stained with H&E. The morphology of the cells was then examined under a microscope and photographed (Nikon, China).

### 2.5. Hoechst 33258 Staining Assay

The cells (1 × 10^6^/well) were seeded into 6-well plates, collected after SSD treatment for 48 h, and fixed with 4% paraformaldehyde for 15 min. Subsequently, the cells were stained with Hoechst 33258 and stored for 5 min in dark conditions at 37°C, and the nuclear morphology of cells was observed under a fluorescence microscope (Carl Zeiss, Germany).

### 2.6. Annexin V/Propidium Iodide (PI) Staining-Based Apoptosis Assay

The Annexin V-FITC Apoptosis Kit was used to quantify cell apoptosis. Cells (1 × 10^6^/well) were seeded in 6-well plates and treated with various concentrations (0, 9, and 15 *μ*M) of SSD for 48 h. The samples were digested with ethylenediaminetetraacetic acid- (EDTA-) free trypsin, washed thrice with phosphate-buffered saline (PBS), thoroughly mixed with a blocking buffer, and stained with Annexin V-FITC conjugate for 10 min and PI for 5 min before fluorescence-activated cell sorting analysis (BD Biosciences, San Jose, CA, USA). Cells were classified as viable (Annexin−/PI−), early apoptotic (Annexin+/PI−), late apoptotic (Annexin+/PI+), and necrotic (Annexin−/PI+).

### 2.7. Western Blotting

Following the SSD treatment for 48 h, the RG-2, U87-MG, U251, and LN-428 cells were digested with EDTA-free trypsin and washed thrice with ice-cold PBS. The cells were lysed in the radioimmunoprecipitation assay lysis buffer (Beyotime, Shanghai, China) containing a protease inhibitor on ice for 40 min. The mixture was centrifuged at 12,000 rpm for 15 min at 4°C. The supernatant was used for bicinchoninic acid quantification, and denaturation was performed in a metal bath for 10 min. Approximately, 25 *μ*g aliquots of protein were loaded in each lane of a polyacrylamide gel and separated using 10%–12% sodium dodecyl sulfate-polyacrylamide gel electrophoresis. The separated proteins were transferred onto polyvinylidene difluoride membranes. The membranes were blocked with 5% nonfat milk at room temperature for 1.5 h. The appropriate primary antibody was added (goat anti-mouse antibody GRP78, CHOP, ATF6, Beclin 1, Caspase-9, and Caspase-3 at 1 : 5,000 dilution; goat anti-rabbit antibody PERK, LC3, PARP, and Caspase-12 at 1 : 2,000 dilution) and incubated overnight at 4°C. On day 2, the membranes were washed thrice (10 min each) with Tris-buffered saline with Tween (TBST), and horseradish peroxidase-labeled secondary antibody (Proteintech Group, Inc.) was added at 1 : 5,000 dilution. The membranes were then incubated for 1.5 h at room temperature. The membranes were again washed thrice (10 min each) with 1 × TBST. After adding an enhanced chemiluminescent substrate (Boster, Wuhan, China), the membrane was exposed and photographed using a UVP gel imager (Gene Company Limited, GBOX). The gray value was obtained using ImageJ software (National Institutes of Health), and the relative protein levels were calculated using the gray value of the *β*-actin internal control as a reference.

### 2.8. ICC

ICC was performed on cells bearing coverslips in the different experimental groups. The following antibodies were used: goat anti-rat GRP78 (1 : 500), goat anti-rat CHOP (1 : 250), goat anti-rabbit PERK (1 : 500), goat anti-rat ATF6 (1 : 500), goat anti-rabbit PARP (1 : 500), goat anti-rabbit Caspase-12 (1 : 500), goat anti-rat Caspase-9 (1 : 500), goat anti-rat Caspase-3 (1 : 500), goat anti-rat Beclin 1 (1 : 500), and goat anti-rabbit LC3 (1 : 250). The color reaction was performed using 3′-diaminobenzidine tetrahydrochloride (Boster, Wuhan, China). Based on the labeling intensity, the staining results were evaluated by three independent researchers and rated as negative (−) when no immunolabeling was observed in the target cells, weakly positive (+) when the labeling was faint, and moderately positive (++) or strongly positive (>++) when the labeling was stronger or distinctly stronger than (++). We followed the methods described by Liu et al. in 2020 [17].

### 2.9. Statistical Analyses

Data are expressed as mean ± standard deviation of triplicate experiments. Statistical analysis and scientific mapping of the experimental data were performed using GraphPad Prism software (version 8.0, GraphPad Software, Inc., La Jolla, CA, USA), and independent *t*-tests were used for pairwise comparisons. Statistical significance was set at a *P* value <0.05.

## 3. Results

### 3.1. SSD Inhibiting the Proliferation of GBM Cells

To determine whether or not SSD inhibited the growth of RG-2, U87-MG, U251, and LN-428 cells, the cell viability rate was assessed using the CCK-8 method. SSD inhibited the proliferation of RG-2, U87-MG, U251, and LN-428 cells. Four cell lines were inoculated in 96-well plates and treated with different concentrations (0, 9, 12, 15, 18, and 21 *μ*M) of SSD for 24, 48, and 72 h. The proliferation rates of RG-2, U87-MG, U251, and LN-428 cells were significantly reduced in a dose-dependent manner (Figure 1(b)). The proliferation of RG-2, U87-MG, and U251 cells was also inhibited in a time-dependent manner compared with that of cells in the control group. Additionally, IC50 of RG-2, U87-MG, U251, and LN-428 cells were 14.22, 15.07, 11.94, and 17.28 *μ*M, respectively. Thus, the sensitivity rankings of the four cell types to SSD were U251 > U87 − MG > RG − 2 > LN − 428 (Figure 1(b)).

### 3.2. Effect of SSD Treatment on GBM Cell Morphology

**Figure 2:**
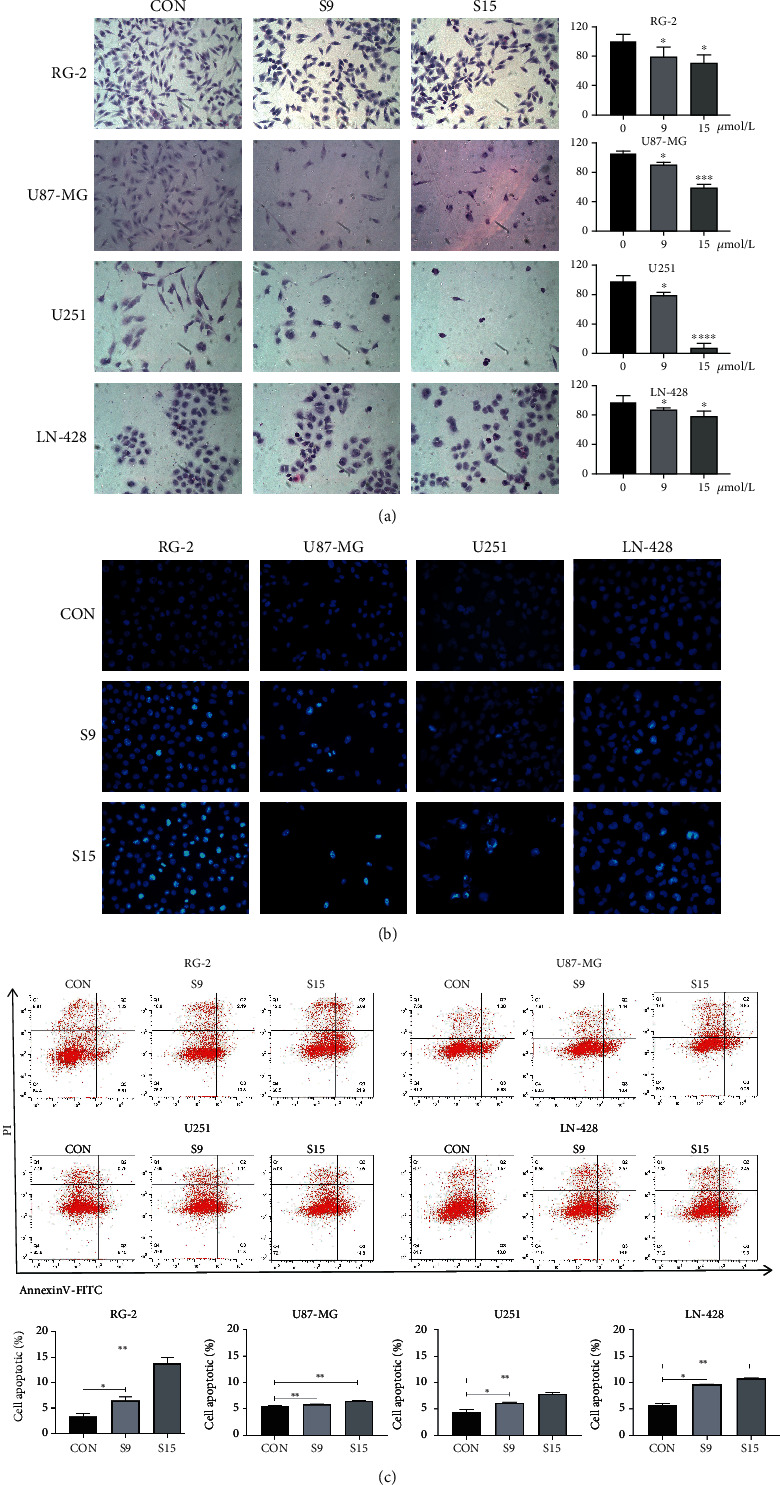
SSD-caused apoptosis in GBM cells. The changes in cell morphology of SSD (9 *μ*M and 15 *μ*M) treatments for 48 h on RG-2, U87-MG, U251, and LN-428 GBM cell lines. (a) H&E morphological staining (20× magnification). (b) Hoechst 33258 fluorescence staining (40× magnification) observe the morphology of apoptosis. (c) Flow cytometry analysis of Annexin V/PI in four cells for apoptosis. ^∗^*P* < 0.05, ^∗∗^*P* < 0.01, and ^∗∗∗^*P* < 0.001 vs. CON group. The error bars indicate standard error.

Changes in the morphology of the four cell lines were examined under an inverted microscope (Nikon, China), and these cells were treated with SSD. Microscopic examination showed that the RG-2, U87-MG, U251, and LN-428 cells adhered to the wall and grew, had a larger volume, and appeared fusiform, triangular, and polygonal in shape compared with the corresponding cells in the control group (Figure 1(c)). However, the adhesion ability of the cells decreased, and the number of suspended cells increased. The cells demonstrated a short spindle or round morphology. Moreover, tiny particles were found in the cell cytoplasm, and the opacity became poor after treatment with different concentrations of SSD for 24, 48, and 72 h, compared with their corresponding presentation in the control group (Figure 1(c)). This phenomenon became more significant as SSD concentration increased. In summary, high and low concentrations (9 *μ*M and 15 *μ*M) of SSD were selected for subsequent experiments. H&E staining revealed a reduction in cell number and distinct alterations in the morphologies of RG-2, U87-MG, U251, and LN-428 cells (Figure 2(a)). The growth of the four GBM cell lines was significantly suppressed after treatment with 15 *μ*M SSD (*P* < 0.01), accompanied by cellular shrinkage, chromatin condensation, and formation of apoptotic bodies (Figure 2(a)).

### 3.3. SSD Treatment Causing Apoptosis in GBM Cells

Hoechst 33258 stain binds to DNA to brighten the apoptotic nuclei. With an increase in the SSD concentration, the number of brightly stained cells increased, particularly in RG-2 cells (Figure 2(b)). The results of flow cytometry based on Annexin V-FITC/PI double staining are shown in Figure 2(c). The proportion of apoptotic cells in control RG-2 cells was 3.83%, which increased to 6.89% and 14.59% after treatment with 9 and 15 *μ*M SSD, respectively, for 48 h (*P* < 0.05). In contrast, the proportions of apoptotic U87-MG, U251, and LN-428 cells were 5.9%, 6.34%, and 9.73% (*P* < 0.01), respectively, after treatment with 9 *μ*M SSD. The proportions of apoptotic U87-MG, U251, and LN-428 cells increased to 6.57%, 7.80%, and 10.87% (*P* < 0.01), respectively, after treatment with 15 *μ*M SSD. These results revealed that SSD induced apoptosis of RG-2, U87-MG, U251, and LN-428 cells in a concentration-dependent manner.

### 3.4. SSD Activating ER Stress-Related Proteins

**Figure 3:**
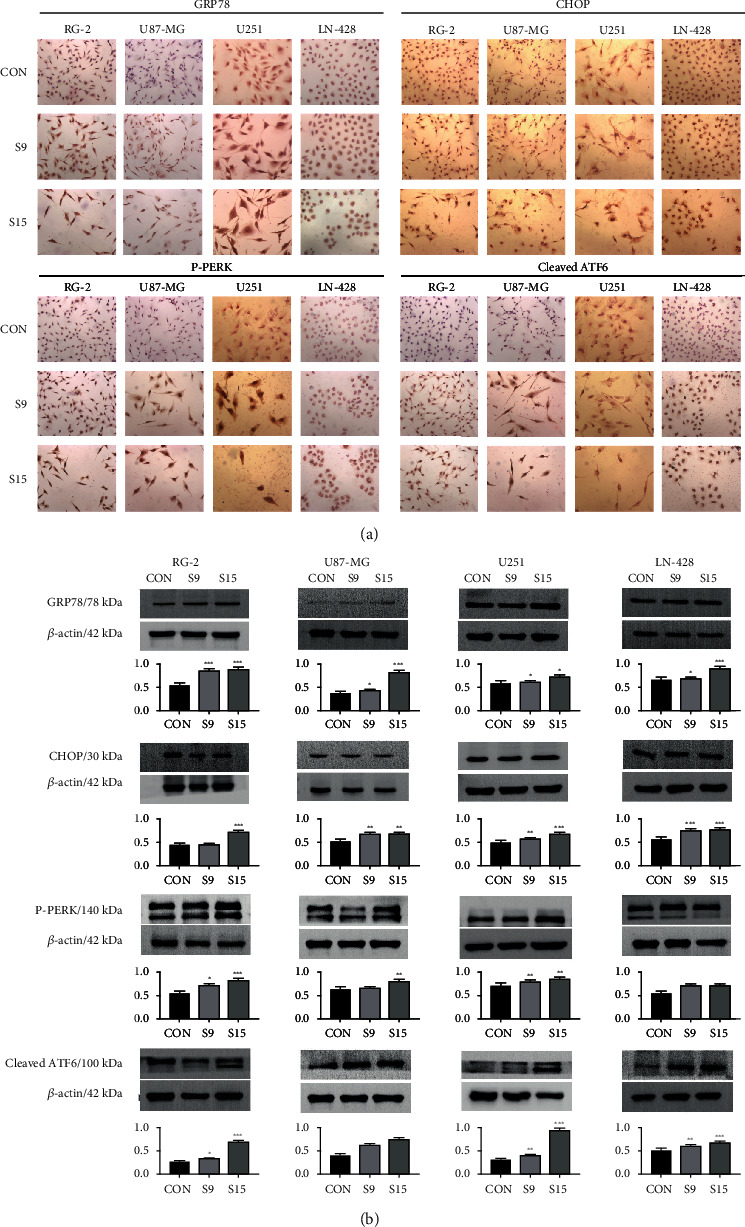
SSD activated ER stress-related proteins. Activated the expression of ER stress-associated proteins GRP78, CHOP, PERK, and ATF6 in RG-2, U87-MG, U251, and LN-428 GBM cell lines treated by SSD. (a) ICC staining (40× magnification). (b) Western blotting. *β*-Actin was used as qualitative and quantitative control. Ratio, the ratio between the levels of the target molecules and that of *β*-actin; ^∗^ with statistical significance (^∗^*P* < 0.05, ^∗∗^*P* < 0.01, and ^∗∗∗^*P* < 0.001 vs. CON group). The error bars, the mean ± standard deviation.

To determine the molecular mechanisms underlying the inhibition of ER stress-induced cell growth by SSD, ER stress-dependent apoptosis-related proteins (GRP78, CHOP, PERK, and ATF6) were examined. ICC revealed remarkably increased production of GRP78, CHOP, phosphorylation-PERK (p-PERK), and cleaved ATF6 in the four GBM cell lines treated with SSD compared with that of control cells (Figure 3(a)). Western blotting showed that the GRP78 expression in RG-2 cells increased by 56.3% after treatment with 9 *μ*M SSD for 48 h and 60.0% after treatment with 15 *μ*M SSD compared with that in the control cells (Figure 3(b)). The level of GRP78 also increased in U87-MG (90.3%), U251 (32.5%), and LN-428 (37.3%) cells after treatment with 15 *μ*M SSD (Figure 3(b)). Moreover, western blotting revealed that the CHOP level in RG-2 cells increased by 64.4% after treatment with 15 *μ*M SSD for 48 h, 32.5% in U87-MG cells after treatment with 15 *μ*M SSD, 34.2% in U251 cells, and 34.8% in LN-428 cells compared with that in the corresponding control cells (Figure 3(b)). After treatment with various concentrations of SSD, the phosphorylation of PERK (p-PERK) and cleavage of ATF6 (cleaved ATF6) increased. The p-PERK levels in RG-2, U87-MG, U251, and LN-428 cells were 46.4%, 23.7%, 18.8%, and 24.7%, respectively, higher than those in normal glioma cells (*P* < 0.01; Figure 3(b)), and the levels of cleaved ATF6 increased by 89.3%, 78.6%, 92.4%, and 29.1% (*P* < 0.01) in RG-2, U87-MG, U251, and LN-428 cells, respectively, when treated with 15 *μ*M SSD for 48 h compared with those in the corresponding control cells.

### 3.5. SSD-Induced Cell Apoptosis via the ER Stress-Dependent Pathway

**Figure 4:**
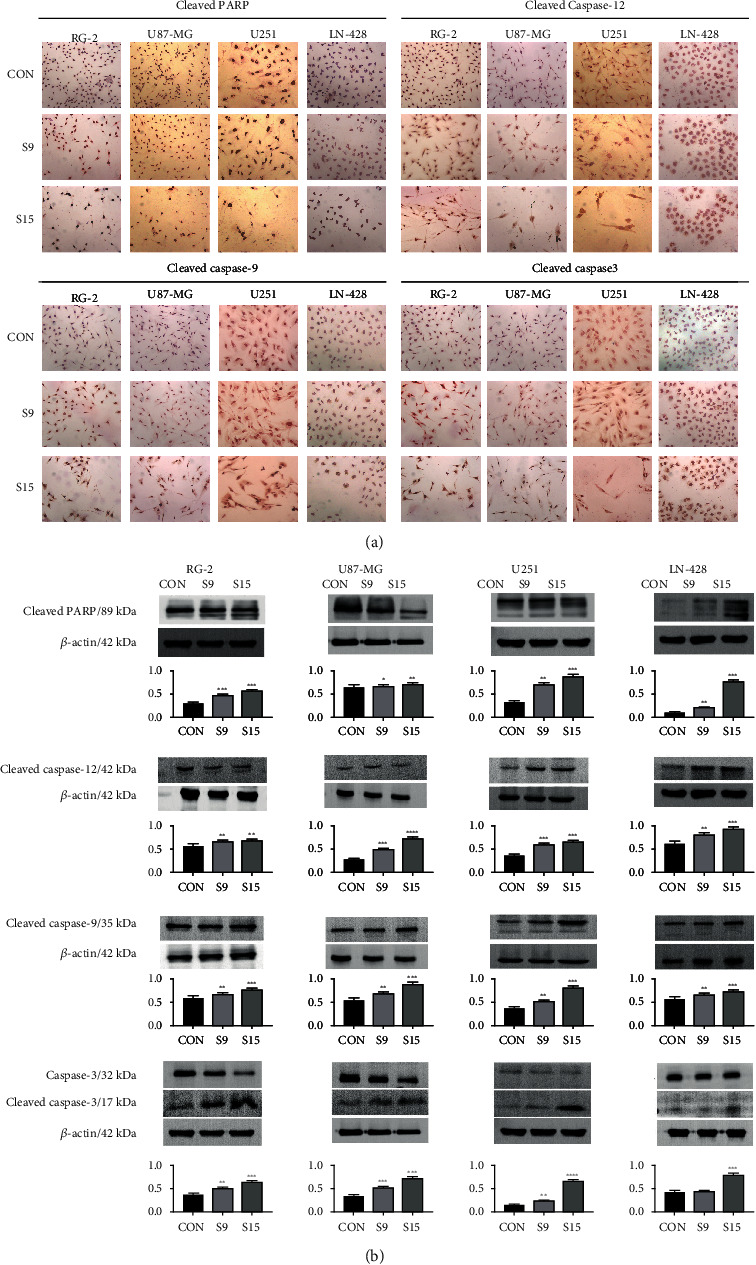
SSD-induced cell apoptosis of GBM cell lines. Cleaved the expression of apoptosis-related proteins PARP, Caspase-12, Caspase-9, and Caspase-3 in RG-2, U87-MG, U251, and LN-428 GBM cell lines treated by SSD. (a) ICC staining (40× magnification). (b) Western blotting. *β*-Actin was used as qualitative and quantitative control. Ratio, the ratio between the levels of the target molecules and that of *β*-actin; ^∗^ with statistical significance (^∗^*P* < 0.05, ^∗∗^*P* < 0.01, and ^∗∗∗^*P* < 0.001 vs. CON group). The error bars, the mean ± standard deviation.

To further illustrate the effect of ER stress induced by SSD on cells, ICC showed that Caspase-12, Caspase-9, and Caspase-3 were expressed in the cytoplasm, and PARP was predominantly localized in the nuclei of four GBM cells after treatment with 9 and 15 *μ*M SSD (Figure 4(a)). Western blotting showed that the expression level of cleaved Caspase-12 in RG-2 cells increased by 17.6% after treatment with 15 *μ*M SSD for 48 h compared with that in the control RG-2 cells. Caspase-9 and Caspase-3 were predominantly localized in the cytoplasm of RG-2 cells, became cleaved Caspase-9 and Caspase-3, and their expression levels increased by 28.1% and 71.4%, respectively, compared with the corresponding levels in the control groups, after treatment with 15 *μ*M SSD for 48 h. The cleaved Caspase-9 and Caspase-3 were also activated in the U87-MG (59.1% and 89.3%, respectively), U251 (90.3% and 98.8%, respectively), and LN-428 (25.8% and 79.8%, respectively) cells after treatment with SSD (9 and 15 *μ*M, respectively) for 48 h compared with that in the corresponding control cells. However, cleaved PARP was expressed in the nucleus, and the expression levels increased by 77.8%, 71.4%, 96.4%, and 98.5% in RG-2, U87-MG, U251, and LN-428 cells, respectively, after treatment with 15 *μ*M SSD for 48 h, compared with the corresponding levels in the control groups (Figure 4(b)). These results indicated that SSD induces cell apoptosis by activating ER stress-dependent apoptosis.

### 3.6. Activation of ER Stress-Dependent Autophagy by SSD

**Figure 5:**
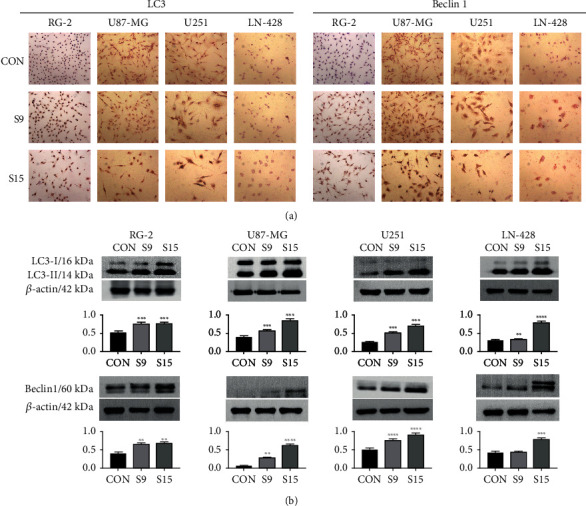
SSD-induced cell autophagy of GBM cell lines. Activated the expression of autophagy-related proteins LC3 and Beclin 1 in RG-2, U87-MG, U251, and LN-428 GBM cell lines treated by SSD. (a) ICC staining (40× magnification). (b) Western blotting. *β*-Actin was used as qualitative and quantitative control. Ratio, the ratio between the levels of the target molecules and that of *β*-actin; ^∗^ with statistical significance (^∗^*P* < 0.05, ^∗∗^*P* < 0.01, ^∗∗∗^*P* < 0.001, and ^∗∗∗∗^*P* < 0.0001 vs. CON group). The error bars, the mean ± standard deviation.

We further investigated ER stress-dependent autophagy proteins and the expression levels of LC3 and Beclin 1 in GBM cells posttreatment. Western blotting and ICC results revealed that after treatment with 15 *μ*M SSD for 48 h, the LC3-II/LC3-I levels increased by 50.6% in RG-2 cells, 67.7% in U87-MG cells, 89.5% in U251 cells, and 80.4% in LN-428 cells, compared with those in the corresponding control groups. Moreover, after treatment with 15 *μ*M SSD, the expression level of Beclin 1 increased by 66.4% in RG-2 cells, 90.2% in U87-MG, 72.8% in U251, and 79.5% in LN-428 cells compared with that in the corresponding control cells (Figures 5(a) and 5(b)). Therefore, these results indicated that autophagy in cells can be significantly induced by SSD-induced ER stress.

## 4. Discussion

GBM is the most common type of malignant brain tumor in adults. It is an aggressive primary intracranial tumor with a poor prognosis. The survival rate of patients with GBM remains poor; the overall 5-year relative survival rate is among the lowest of all cancer types (4%–5%) [18]. Despite the treatment options available for glioblastoma, including surgical treatment, radiotherapy, and chemotherapy, patients have a median survival time of only 15 months [19]. Over the past 30 years, the survival rates of patients with GBM have shown only slight improvement. Currently, temozolomide [20] and tumor treatment field (TTF) [21] are used to treat GBM. Noninvasive devices from Novocure GmbH have demonstrated clinical efficacy and improved results. However, owing to the development of drug resistance to temozolomide [22] and the high cost of TTF, a new drug with low toxicity and high efficiency is urgently required.

SSD has been isolated from RB plants and is a potent compound with several important biological properties, such as antioxidant activity, anti-inflammatory activity, and cancer inhibition [23, 24]. In this study, SSD was selected as an anti-GBM model drug, and its efficacy in treating GBM was evaluated. The results revealed that SSD decreased cell viability in a dose-dependent manner, and cells incubated with SSD showed obvious signs of early apoptosis compared with control cells. The effects of SSD on the proliferation of RG-2, U87-MG, U251, and LN-428 cells were determined using a series of CCK-8 experiments. Apoptotic bodies were observed using H&E staining. The formation of apoptotic corpuscles is considered the most direct evidence of apoptosis [25]. Almost all living organisms contain genetic information in DNA. Many anticancer drugs, such as cisplatin and camptothecin, damage DNA and cause apoptosis [26, 27]. Hoechst 33258 staining revealed DNA damage [28]. Based on the staining results, the cell growth rate significantly decreased after SSD treatment, indicating its excellent antiproliferative properties.

When ER stress persists, the unfolded protein response is insufficient to remove accumulated unfolded proteins or damaged organelles, and autophagy is activated [29]. When ER stress is too strong or lasts too long, overactivation of autophagy eventually leads to apoptosis [30]. GRP78 overexpression and UPR components have been implicated in the development of malignant gliomas with aggressive phenotypes, whereas ER stress predisposes glioma cells to undergo apoptosis [31–33]. Whether or not ER stress plays a role in the effects of SSD on GBM cells is unknown, although cervical cancer cells are sensitive to Saikosaponin A [10]. Therefore, ER stress marker proteins GRP78 and CHOP were selected. Western blotting and ICC results showed that the expression levels of GRP78 and CHOP were markedly enhanced after the SSD treatment. Furthermore, we analyzed the ER stress downstream pathway proteins PERK and ATF6 and observed that the PERK protein was phosphorylated, while ATF6 was cleaved, indicating that ER stress was activated by the SSD treatment. However, the ER stress pathway also plays an important role in apoptosis [34]. ER stress can be caused by various toxic injuries, eventually resulting in cell apoptosis. If ER activation persists, cells eventually initiate Caspase-12-dependent apoptosis [16]. Caspase-9 is the initiator caspase, while Caspase-3 is the executioner caspase that causes programmed cell death when activated [35]. PARP is a DNA repair enzyme, cutting substrate of caspases, and core member of apoptosis [36] that plays an important role in DNA damage repair and apoptosis. Therefore, Caspase-12, Caspase-9, Caspase-3, and PARP were analyzed in this study. Western blotting and ICC revealed that Caspase-12, Caspase-9, and Caspase-3 were cleaved, and their protein expression levels were increased in the cytoplasm after the SSD treatment. PARP was expressed in the nucleus and cleaved after the SSD treatment in all four-cell lines. These results indicated that SSD treatment induced cell apoptosis by activating the ER stress-dependent pathway.

ER stress is widely accepted as the most potent trigger of autophagy of many tumor cell types [37]. ATF6 is a crucial sensor of ER stress, and its activation can induce autophagy [38]. Autophagy and apoptosis are two self-degradation mechanisms in multicellular organisms. The difference between autophagy and apoptosis is that autophagy allows the cell to survive stress, while apoptosis does not allow the cell to survive. Autophagy deficiency in apoptosis-impaired tumor cells leads to enhanced genomic instability and tumorigenesis [39, 40]. As a result of SSD-induced GBM inhibition, activation of an ER stress-dependent autophagy mechanism might have occurred. Western blotting and ICC experiments were performed to verify the expression of the autophagy marker proteins LC3-II/I and Beclin 1. The results revealed that the levels of LC3-II/I and Beclin 1 were elevated by the SSD treatment of GBM cells, and the expression signal was proportional to the SSD concentration, indicating that the levels of autophagy increased after the SSD treatment. These results indicated that ER stress-dependent autophagy was activated by the SSD treatment.

## 5. Conclusions

**Figure 6:**
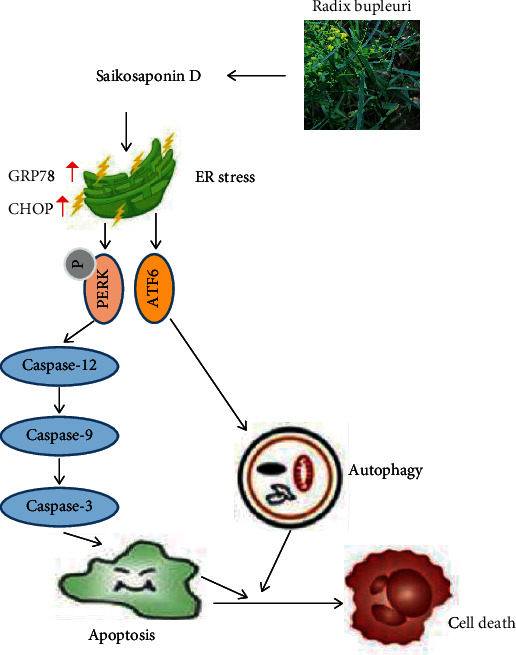
Schematic diagram depicting the anti-GBM mechanism of SSD in GBM cell lines.

SSD inhibited the proliferation of RG-2, U87-MG, U251, and LN-428 cells, thereby exerting an anti-GBM effect. Moreover, the anti-GBM mechanism of SSD was elucidated. SSD caused DNA damage in GBM cells and induced cell apoptosis and autophagy by activating the ER stress-signaling pathway, ultimately inhibiting the growth of GBM cells (Figure 6). Therefore, SSD may be considered a novel therapeutic option for gliomas. The results of this study lay a foundation for further development of SSD and provide new insights into the treatment of GBM.

## Data Availability

All data included in this study are available upon request by contact with the first author.
